# Identification and integrated analysis of lncRNAs and miRNAs in IPEC-J2 cells provide novel insight into the regulation of the innate immune response by PDCoV infection

**DOI:** 10.1186/s12864-022-08722-2

**Published:** 2022-07-04

**Authors:** Shan Jiang, Jianfei Chen, Xiuli Li, Weike Ren, Fengxiang Li, Ting Wang, Cheng Li, Zhimin Dong, Xiangxue Tian, Li Zhang, Lili Wang, Chao lu, Jingjing Chi, Li Feng, Minghua Yan

**Affiliations:** 1 Tianjin Institute of Animal Husbandry and Veterinary Medicine, Tianjin Academy of Agricultural Sciences, Tianjin, 300381 China; 2 Tianjin Observation and Experimental Site of National Animal Health, Tianjin, 300381 China; 3 State Key Laboratory of Veterinary Biotechnology, Harbin Veterinary Research Institute, Chinese Academy of Agricultural Sciences, Harbin, 150069 China; 4 Ministry of Education Key Laboratory of Pollution Processes and Environmental Criteria, College of Environmental Science and Engineering, Nankai University, Tianjin, 300071 China; 5 Institute of Pathogenic Microorganism and College of Bioscience and Engineering, Jiangxi Agricultural University, Nanchang, 330045 Jiangxi China

**Keywords:** PDCoV, miRNA, lncRNA, Pathogenesis, Host defense response, Intestinal porcine epithelial cells, Li Feng and Minghua Yan contributed equally to this work.

## Abstract

**Background:**

Noncoding RNAs (ncRNAs), including microRNAs (miRNAs) and long noncoding RNAs (lncRNAs), are pivotal regulators involved in the pathogenic mechanism of multiple coronaviruses. Porcine deltacoronavirus (PDCoV) has evolved multiple strategies to escape the innate immune response of host cells, but whether ncRNAs are involved in this process during PDCoV infection is still unknown.

**Results:**

In this study, the expression profiles of miRNAs, lncRNAs and mRNAs in IPEC-J2 cells infected with PDCoV at 0, 12 and 24 hours postinfection (hpi) were identified through small RNA and RNA sequencing. The differentially expressed miRNAs (DEmiRNAs), lncRNAs (DElncRNAs) and mRNAs (DEmRNAs) were screened from the comparison group of IPEC-J2 cells at 0 and 12 hpi as well as the comparison group of IPEC-J2 cells at 12 and 24 hpi. The target genes of these DEncRNAs were predicted. The bioinformatics analysis of the target genes revealed multiple significantly enriched functions and pathways. Among them, the genes that were associated with innate immunity were specifically screened. The expression of innate immunity-related ncRNAs and mRNAs was validated by RT–qPCR. Competing endogenous RNA (ceRNA) regulatory networks among innate immunity-related ncRNAs and their target mRNAs were established. Moreover, we found that the replication of PDCoV was significantly inhibited by two innate immunity-related miRNAs, ssc-miR-30c-3p and ssc-miR-374b-3p, in IPEC-J2 cells.

**Conclusions:**

This study provides a data platform to conduct studies of the pathogenic mechanism of PDCoV from a new perspective and will be helpful for further elucidation of the functional role of ncRNAs involved in PDCoV escaping the innate immune response.

**Supplementary Information:**

The online version contains supplementary material available at 10.1186/s12864-022-08722-2.

## Introduction

Porcine deltacoronavirus (PDCoV) is a new porcine enteropathogenic virus. It causes watery diarrhea, dehydration, and high mortality in newborn piglets, which results in significant economic losses to the swine industry [1]. PDCoV is an enveloped RNA virus with a positive-stranded RNA genome that belongs to the genus *Deltacoronavirus* within the family *Coronaviridae* [2]. The full genome of PDCoV is approximately 25.4 kb in length and arranged in the following order: 5′ untranslated region (UTR), open reading frame 1a and b (ORF1ab), spike (S), envelope (E), membrane (M), non-structural gene 6 (NS6), nucleocapsid (N), non-structural gene 7 (NS7), and 3′ UTR [2]. PDCoV was initially reported in a molecular surveillance study in Hong Kong (China) in 2012, and then emerged in Ohio (USA) in 2014 [3, 4]. Subsequently, it spread to many countries including Thailand, South Korea, Canada, and China [5–9]. Currently, PDCoV is one of the main viruses that causing porcine diarrhea worldwide, and there are still no commercial vaccines or specific drugs for the prevention and treatment of PDCoV [10]. Therefore, it is essential to explore the pathogenic mechanism of PDCoV, which will provide a foundation for the research and development of vaccines and antiviral drugs to prevent and control PDCoV in the future.

Non-coding RNAs (ncRNAs) are a group of RNAs that have no protein-coding potential and are involved in various biological processes. According to the transcript length, ncRNAs can be divided into long noncoding RNAs (lncRNAs) and small RNAs [11]. The lncRNA is a type of long transcript with more than 200 nucleotides, and it is recognized as a transcriptional regulator with *cis* or *trans* regulation [12]. MicroRNAs (miRNAs) are small RNAs (< 200 nucleotides) with a length range of 19–24 nucleotide [13]. They perform their function by regulating gene expression posttranscriptionally through transcript degradation and/or translational inhibition. Viral infection triggers changes in the lncRNA and miRNA expression profiles of host cells, regulating cellular gene expression and strongly affecting influence viral replication [14, 15]. Intestinal porcine epithelial cell line J2 (IPEC-J2) cells, a non-transformed, stable small intestinal columnar epithelial cell line, was isolated from the midjejunal epithelium of a neonatal unsuckled piglet [16, 17]. It is useful to characterize the interactions of enterocytes with enteric viruses in vitro because of the significant physiologic and morphologic similarities to enterocytes in vivo. To date, IPEC-J2 cells have been used as target cells of various porcine enteric coronaviruses, such as porcine epidemic diarrhea virus (PEDV), transmissible gastroenteritis virus (TGEV) and PDCoV [16, 18, 19], and the lncRNA profiles of intestinal tissues of piglets and ST cells infected by PDCoV have been explored [20, 21]. Nevertheless, the alterations in lncRNA and miRNA profiles in IPEC-J2 cells induced by PDCoV are still unclear.

The innate immune system is the first line of host cell defence against pathogens. lncRNAs and miRNAs have been identified as key regulators in the innate immune response. At present, many studies have reported that PDCoV has evolved several strategies to enhance replication by escaping the innate immune response [22–29]. However, the regulatory mechanism of ncRNAs participating in the innate immune evasion of PDCoV is still unclear.

In this study, the lncRNAs, miRNAs and mRNAs of IPEC-J2 cells infected with PDCoV at 0, 12 and 24 hpi were identified by RNA sequencing and small RNA sequencing. An integrative bioinformatics analysis combining the sequencing of the cell samples was performed, and innate immunity-related findings were especially well represented, providing valuable dataset for further investigating the regulatory role of cellular ncRNAs in the innate immune evasion of PDCoV.

## Results

### Proliferation characteristics of PDCoV in IPEC-J2 cells

**Fig. 1:**
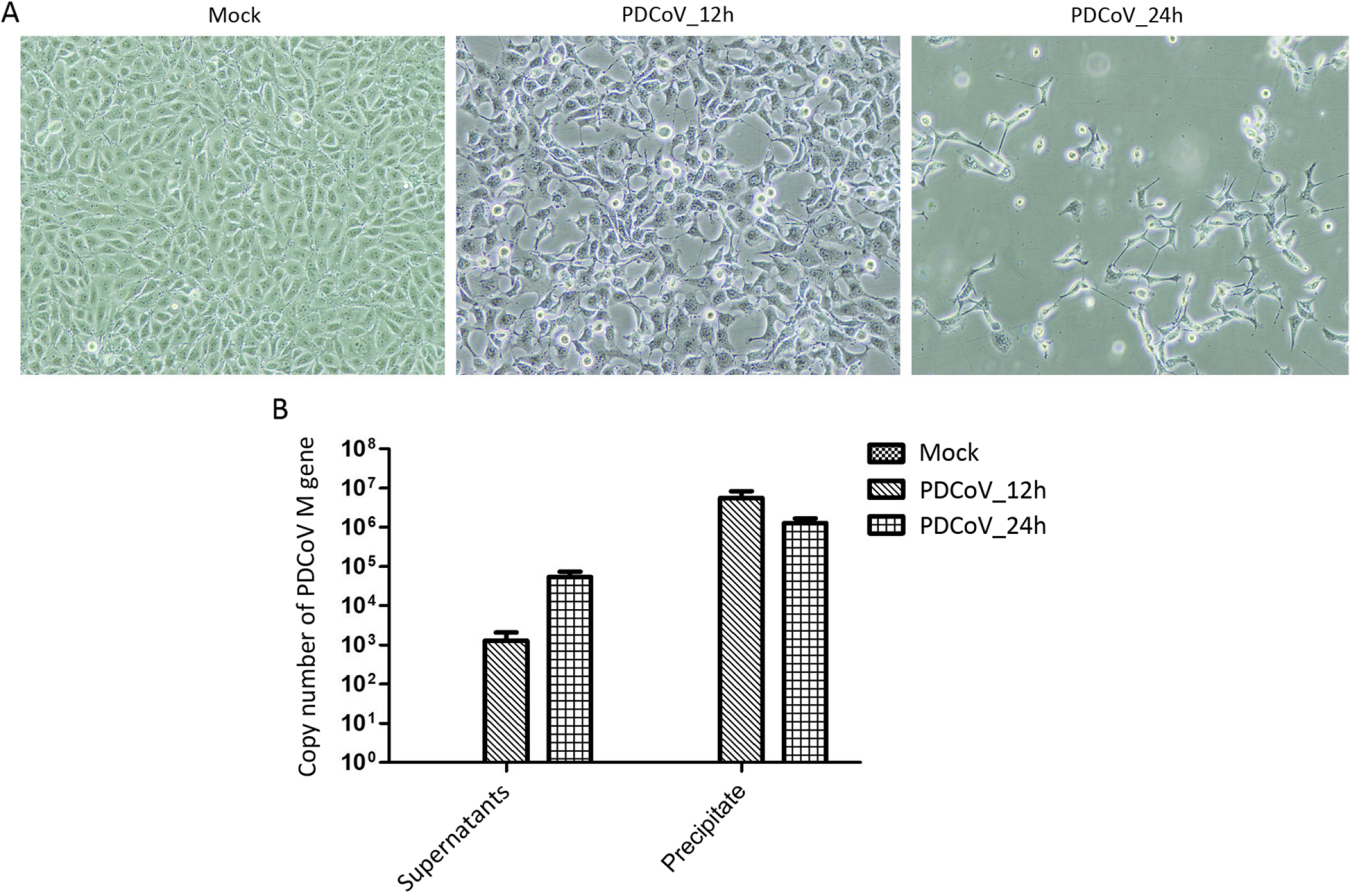
Proliferation characteristics of PDCoV in IPEC-J2 cells. **A** Morphological changes in IPEC-J2 cells at different time points after PDCoV infection. **B** RT-qPCR result of PDCoV M gene in supernatant and precipitate of IPEC-J2 cells at different post-infection time points

Monolayer IPEC-J2 cells were inoculated with the PDCoV TJ_1_ strain at multiplicity of infection (MOI) of 1.0.. Cytopathic effects (CPEs) of cells at 12 hpi consisted of enlargement and rounding cell nuclei as well as cell shrinkage and detachment. At 24 hpi, the majority of the cells had detached and disintegrated, and the residual adherent cells were longer and displayed a spindle shape (Fig. 1A). The copy number of the PDCoV M gene in the cell supernatant and precipitate at different postinfection time points was determined, and the results showed that the virus continuously proliferated in IPEC-J2 cells within 24 h after inoculation, and that the virus in the cells was gradually released into the cell supernatant due to the detachment and disintegration of IPEC-J2 cells (Fig. 1B).

### RNA sequencing and identification of mRNA and lncRNA

To investigate the lncRNA profile and mRNA profile of IPEC-J2 cells during PDCoV infection, cDNA libraries were established from IPEC-J2 cells infected with PDCoV at 0, 12 and 24 hpi (hereafter called Mock, PDCoV_12h, and PDCoV_24h). An average of approximately 754,035,282 raw reads were obtained by RNA sequencing. Low-quality reads and reads with adapters or unknown nucleobases were filtered out, and approximately 752,270,576 high-quality clean reads remained. The percentage of clean reads in each sample ranged from 99.55% to 99.87%. These clean reads were successfully mapped to a swine reference genome (NCBI genome database: *Sus scrofa* 11.1). The transcripts were assembled by StringTie based on the genome comparison results. According to the characteristics of lncRNAs, a total of 7221 transcripts with length ≥ 200 bp and number of exons ≥2 were further obtained. Then, the coding potential of these transcripts were predicted via CPC2 and CNCI software [30, 31]. The new transcripts without coding potential were high-confidence lncRNAs. Ultimately, 11,153 lncRNAs without coding potential, including 1871 novel lncRNAs, were identified. In addition, a total of 20,737 mRNAs, including 71 novel mRNAs, were identified. An overview of the RNA sequencing data is presented in Additional file 1: Table S1–1 and S1–2.

### Small RNA sequencing and identification of miRNA

Through small RNA sequencing, a total of 116,671,318 raw reads were generated from 9 groups of IPEC-J2 cells infected with PDCoV at three different time points. After removing the low-quality reads, including adaptor dimers and reads less than 18 nt, a total of 110,315,356 clean reads remained. The percentage of clean reads ranged from 92.1 to 95.98%. Length distributions of clean reads showed that the majority of the reads were in the range of 21–24 nt. The clean reads were first annotated against the GenBank and Rfam to remove rRNA, scRNA, snoRNA, snRNA, and tRNA sequences. Subsequently, the remaining reads were further mapped to the porcine reference genome (NCBI genome database: *Sus scrofa* 11.1). By removing the reads generated from mRNA degradation as well as the reads mapped to repeat sequences. Eventually, 2400 miRNAs were successfully identified, including 345 existed miRNAs, 930 known miRNAs and 1125 novel miRNAs. An overview of the small RNA sequencing data is presented in Additional file 2: Table S2–1 and S2–2.

### Identification of differentially expressed lncRNAs, mRNAs and miRNAs

To explore the lncRNAs, mRNAs, and miRNAs of IPEC-J2 cells infected with PDCoV at different postinfection times, the expression of all identified lncRNAs, mRNAs, and miRNAs was compared for the IPEC-J2 cell group at 0 and 12 hpi (Mock-vs-PDCoV_12h) and the IPEC-J2 cell group 12 and 24 hpi (PDCoV_12h-vs-PDCoV_24h). Differentially expressed lncRNAs (DElncRNAs) (FDR < 0.05 and a│log2 (fold change)│ > 1), mRNAs (DEmRNAs) (FDR < 0.05 and a│log2 (fold change)│ > 1), and miRNAs (DEmiRNAs) (*P* value < 0.05 and a│log2 (fold change)│ > 1) were identified.

**Fig. 2:**
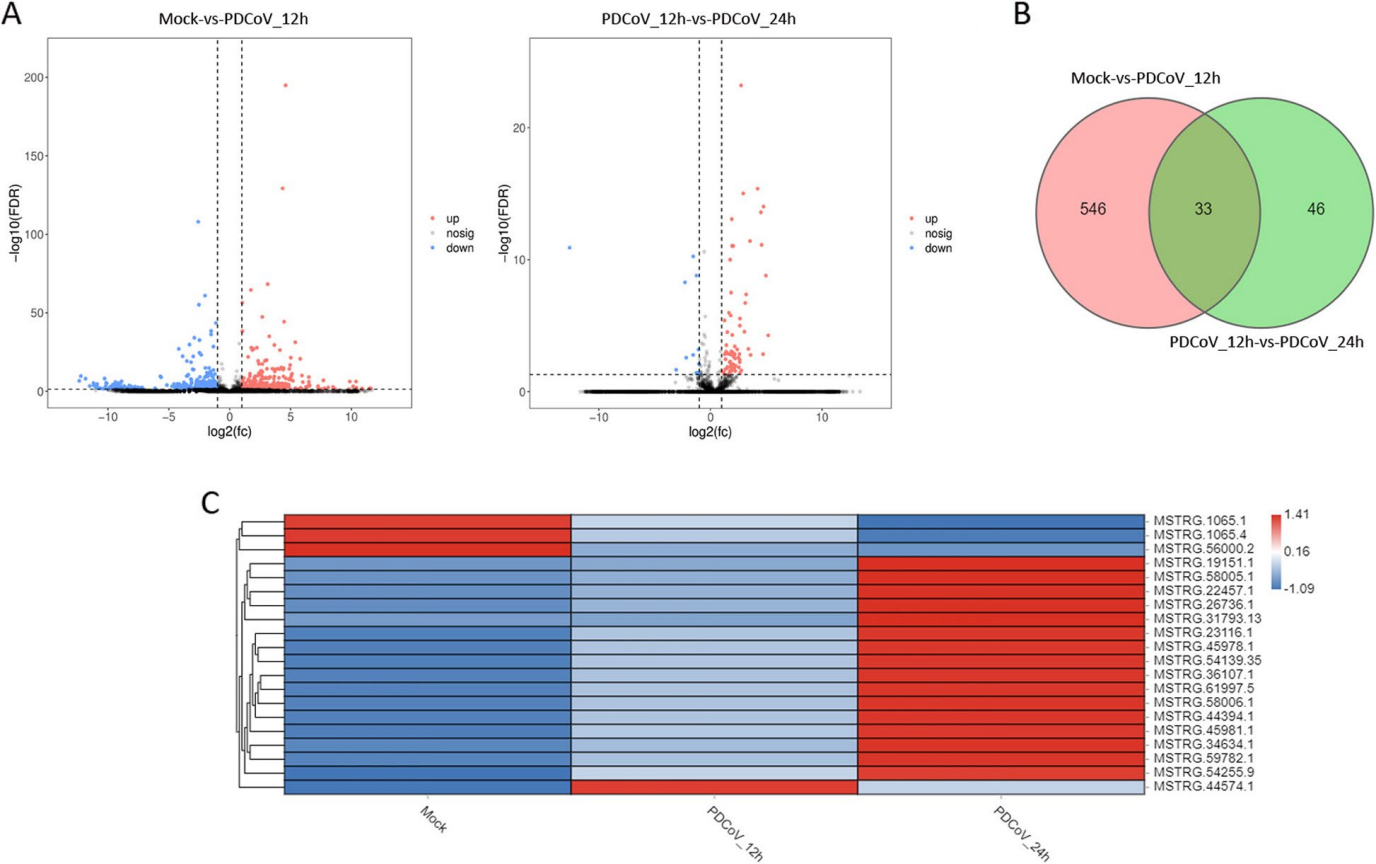
DElncRNAs identified in Mock-vs-PDCoV_12h and PDCoV_12h-vs-PDCoV_24h. **A** The number of DElncRNAs identified in the two comparison groups. **B** The number of DElncRNAs common to the two comparison groups. **C** Hierarchic clustering analyses of the DElncRNAs common to the two comparison groups. Red indicates higher expression, and blue indicates lower expression

**Fig. 3:**
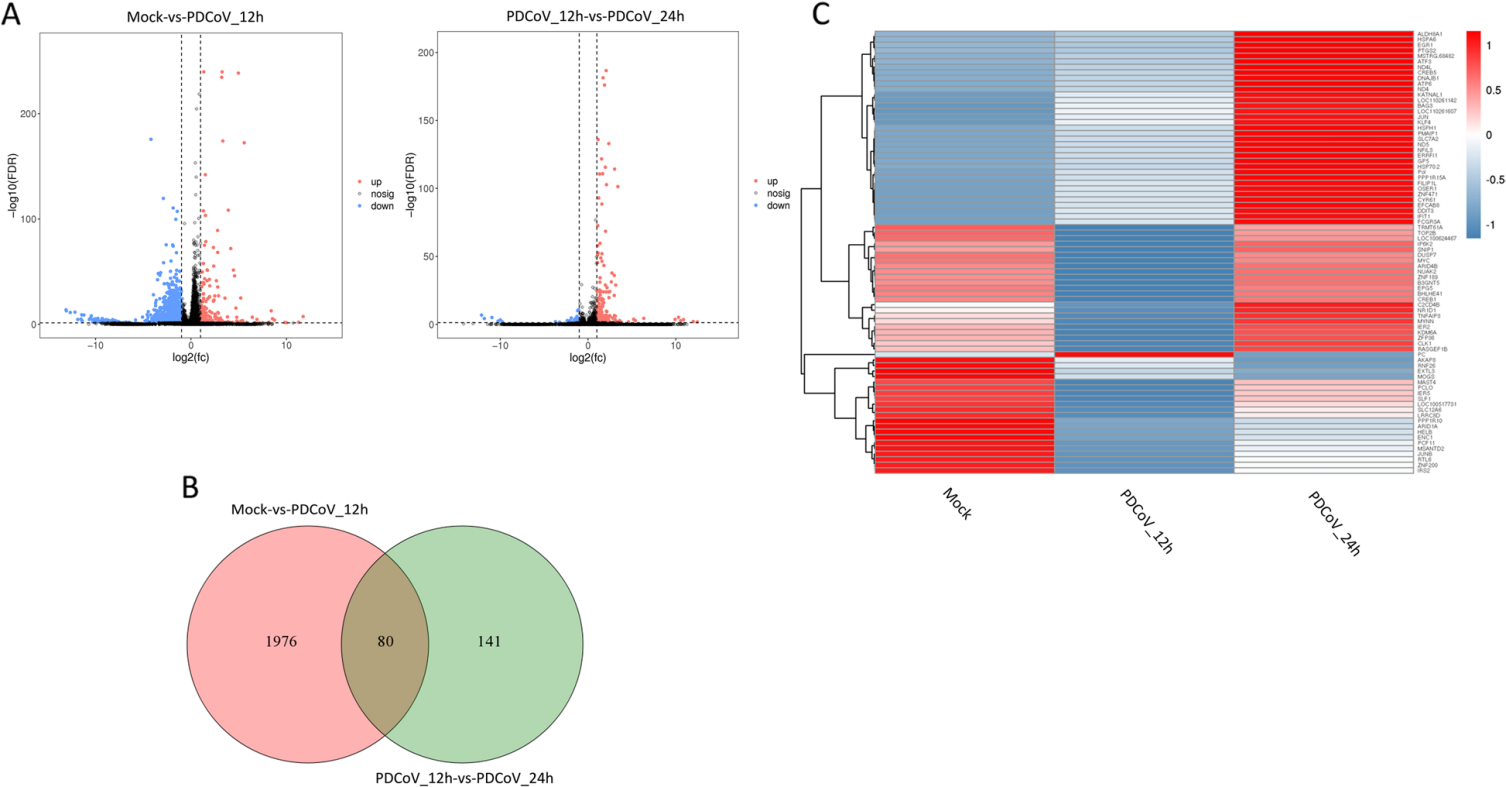
DEmRNAs identified in Mock-vs-PDCoV_12h and PDCoV_12h-vs-PDCoV_24h. **A** The number of DEmRNAs identified in the two comparison groups. **B** The number of DEmRNAs common to the two comparison groups. **C** Hierarchic clustering analyses of the DEmRNAs common to the two comparison groups. Red indicates higher expression, and blue indicates lower expression

**Fig. 4:**
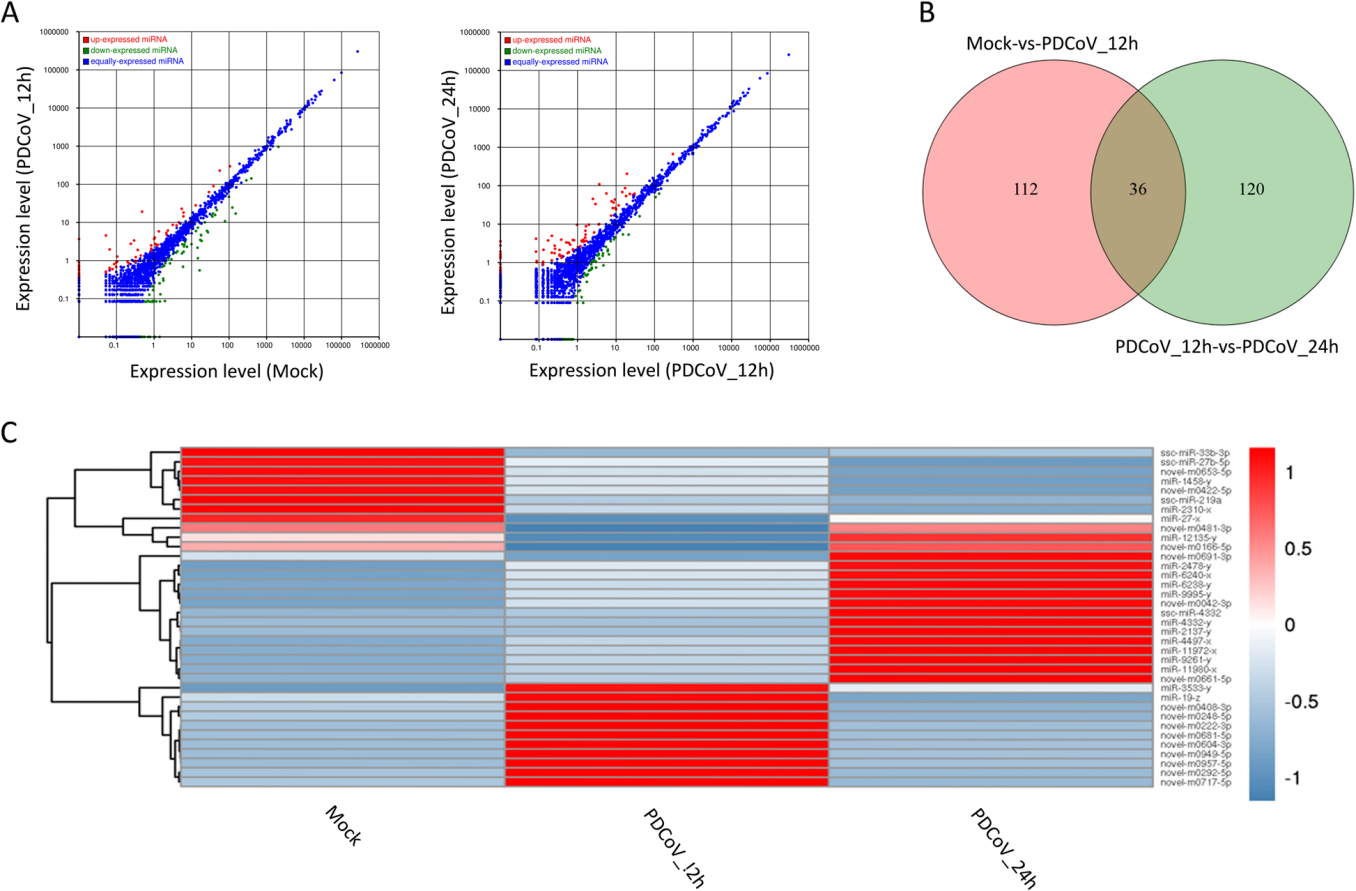
DEmiRNAs identified in Mock-vs-PDCoV_12h and PDCoV_12h-vs-PDCoV_24h. **A** The number of DEmiRNAs identified in the two comparison groups. **B** The number of DEmiRNAs common to the two comparison groups. **C** Hierarchic clustering analyses of the DEmiRNAs common to the two comparison groups. Red indicates higher expression, and blue indicates lower expression

In total, 579 (262 upregulated and 317 downregulated) and 79 lncRNAs (67 upregulated and 12 downregulated) were differentially expressed in Mock-vs-PDCoV_12h and PDCoV_12h-vs-PDCoV_24h, respectively (Fig. 2A, Additional file 3: Table S3–1 and S3–2). There were 33 DElncRNAs common to the two comparison groups (Fig. 2B and C). A total of 2056 (222 upregulated and 1834 downregulated) and 221 mRNAs (181 upregulated and 40 downregulated) were differentially expressed in Mock-vs-PDCoV_12h and PDCoV_12h-vs-PDCoV_24h, respectively (Fig. 3A, Additional file 3: Table S3–3 and S3–4). Eighty DEmRNAs were common to the two comparison groups (Fig. 3B and C). As shown in Fig. 4A, 148 (60 upregulated and 88 downregulated) and 156 miRNAs (82 upregulated and 74 downregulated) were differentially expressed in Mock-vs-PDCoV_12h and PDCoV_12h-vs-PDCoV_24h, respectively (Additional file 3: Table S3–5 and S3–6). From these, 36 DEmiRNAs were common to the two comparison group (Fig. 4B and C).

### Target prediction for DElncRNAs and DEmiRNAs

All of the DElncRNAs were selected to perform *cis*- or *trans*-target gene prediction. We used BEDTools.v2.1.2 software to search for *cis*-target genes from all identified mRNAs located within 10 kb upstream and downstream of DElncRNAs. The *trans* target genes were predicted from DElncRNAs and DEmRNAs by correlation analysis between lncRNAs and mRNAs [32]. Finally, 364 and 101,094 DElncRNA *cis*-target pairs were identified in Mock-vs-PDCoV_12h, and 34 and 2902 DElncRNA *trans*-target pairs were identified in the other comparison group (Additional file 4: Table S4–1, Table S4–2, Table S4–3, and Table S4–4).

From the DEmRNAs, the potential target genes of DEmiRNAs in the two comparison groups were predicted based on miRNA-mRNA negative correlation in expression using three prediction algorithms (TargetScan, miRanda, and RNAhybrid). Finally, 96 and 50 DEmiRNAs with have one or more targets were screened from Mock-vs-PDCoV_12h and PDCoV_12h-vs-PDCoV_24h, respectively (Additional file 5: Table S5–1 and Table S5–2).

Innate immunity constitutes a crucial defence response of host cells against viral infection. The pig immune system is very similar to that of humans [33, 34]. To screen the DElncRNAs and DEmiRNAs that may regulate the innate immune response of IPEC-J2 cells during PDCoV infection, the target genes of these two ncRNAs were annotated to the innate immune gene list of the InnateDB database (https://www.innatedb.ca/annotatedGenes.do?type=innatedb), which provides curated human genes associated with the innate immune response. In the comparison of Mock and PDCoV_12h, a total of 390 DElncRNAs shared potential innate immune target genes (21 DElncRNAs had *cis*-regulatory target genes, and 369 DElncRNAs had *trans*-regulatory target genes) (Additional file 6: Table S6–1 and S6–2), while in another comparison group, a total of 62 DElncRNAs had potential innate immune target genes (0 DElncRNAs had *cis*-regulatory target genes, and 62 DElncRNAs had *trans*-regulatory target genes) (Additional file 6: Table S6–3). In terms of miRNAs, 24 and 18 DEmiRNAs had potential innate immune target genes, respectively (Additional file 7: Table S7–1 and S7–2).

### RT-qPCR validation

**Fig. 5:**
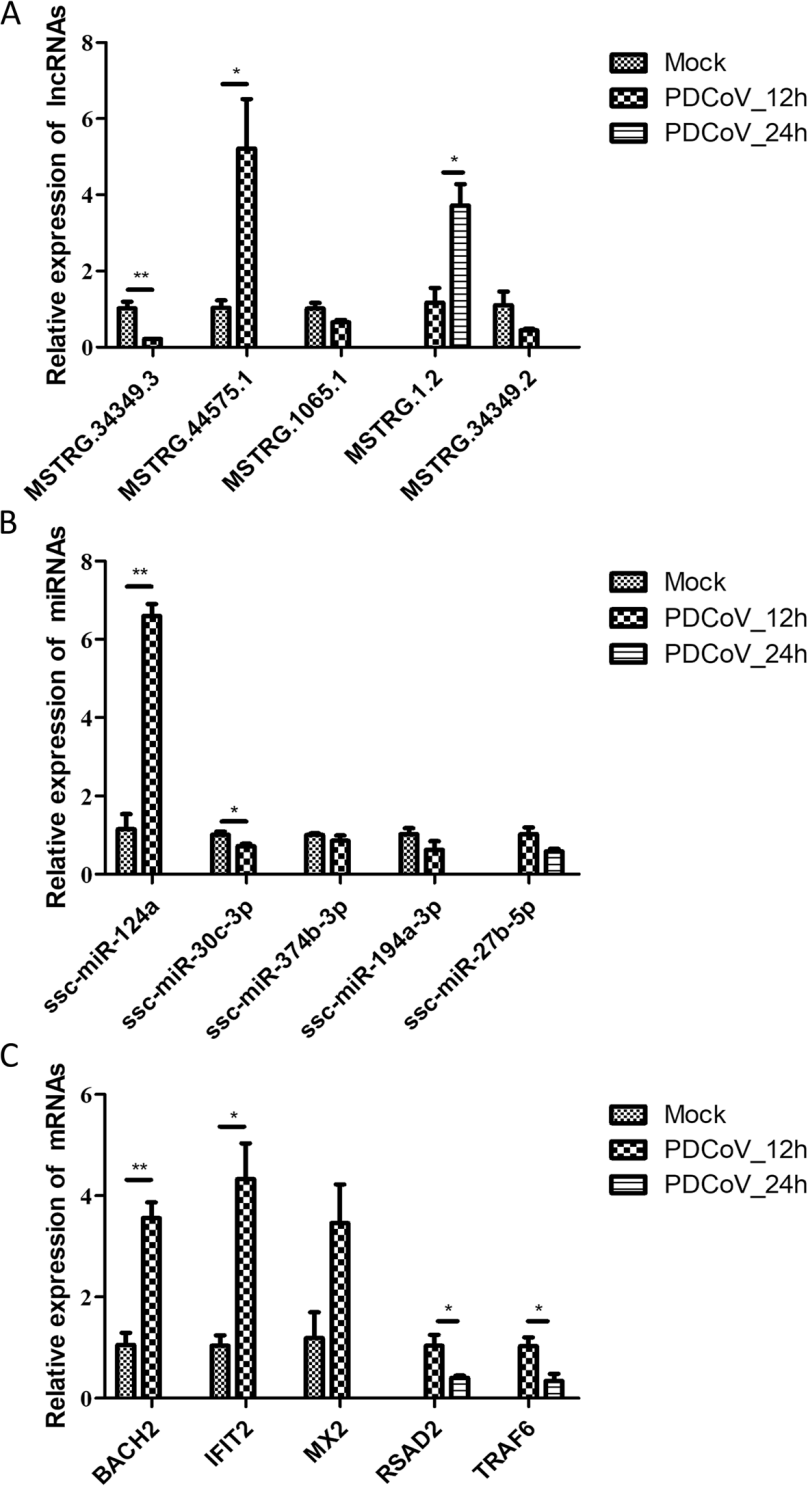
Validation of innate immunity-related DElncRNAs, DEmRNAs and DEmiRNAs expression which performed by RT-qPCR. Data are shown as mean ± SEM, *n* = 3. *P < 0.05, **P < 0.01, ***P < 0.001

To explore the reliability of the high-throughput sequencing data and provide a reference for further investigating the functional roles of ncRNAs in regulating the innate immune response during PDCoV infection, we randomly selected several innate immunity-related DEmiRNAs, DElncRNAs and DEmRNAs to validate their relative expression in the two comparison groups. The results of RT-qPCR were consistent with the sequencing data (Fig. 5).

### Function and pathway enrichment analysis of targets of DElncRNAs

**Fig. 6:**
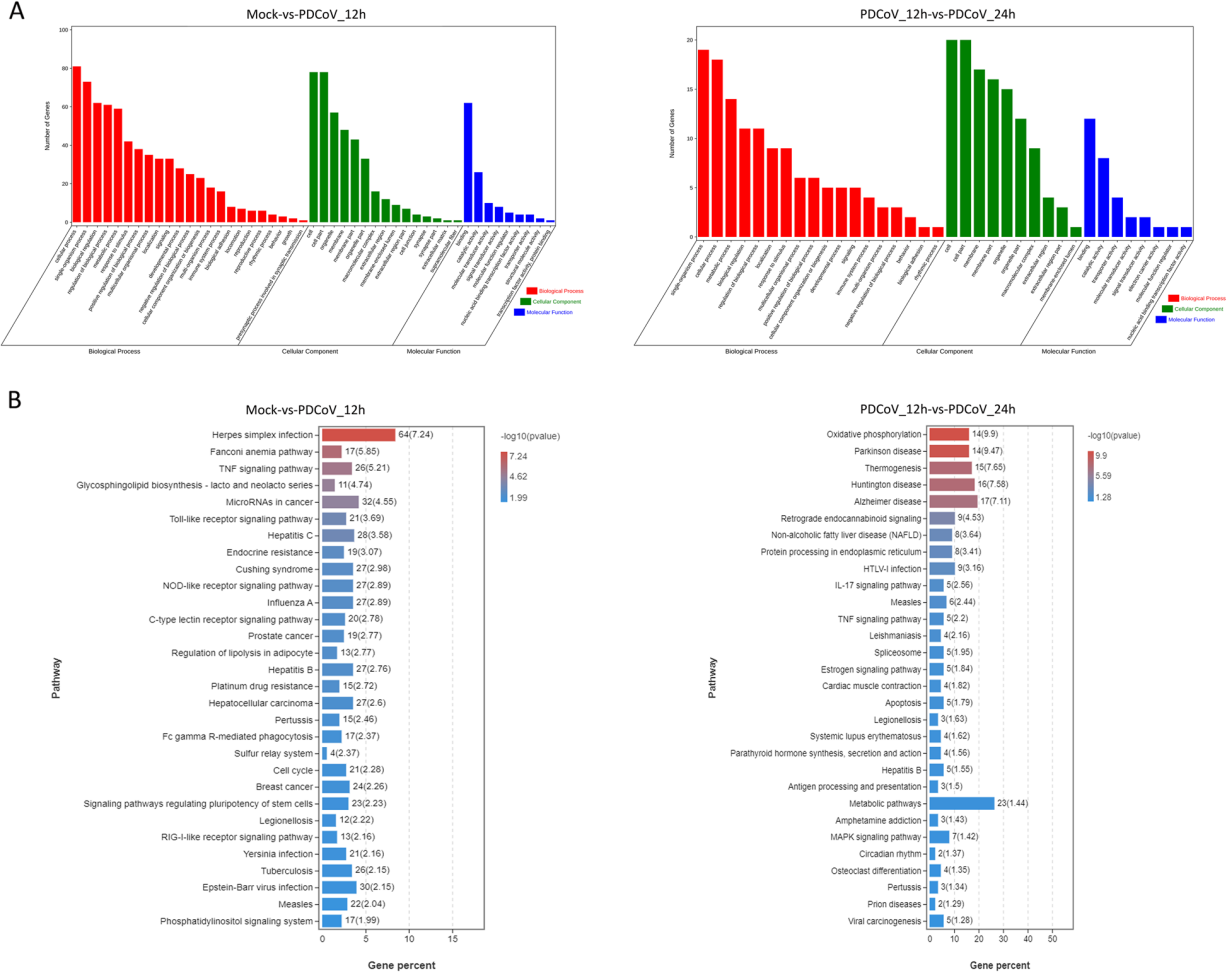
GO function classification and KEGG pathway enrichment results of the target genes of DElncRNAs in Mock-vs-PDCoV_12h and PDCoV_12h-vs-PDCoV_24h. **A** GO function classification of the target genes of DElncRNAs in the two comparison groups. **B** The top 30 KEGG pathways which enriched in the two comparison groups

To further explore the functions of DElncRNAs that were primarily regulated by PDCoV at different postinfection times, GO function enrichment analysis of the predicted targets of DElncRNAs was performed. The target genes were first annotated into the GO database (http://geneontology.org/) and were divided into three categories, including *Biological Process*, *Cellular Component*, and *Molecular Function* (Fig. 6A). Among them, most of the functions belonged to *Biological Process*. The functions in the *Biological Process* category in the two comparison groups were almost the same, which included the regulation of biological processes of cellular immunity in response to external stimulation (immune system process and response to stimulus). The DElncRNA target genes in Mock-vs-PDCoV_12h were significantly enriched in 226 GO terms (*P* < 0.05) (Additional file 8: Table S8–1), which included multiple innate immunity-related terms (Table 1). The enriched GO terms were related to the regulation of the I-kappaB kinase/NF-kappaB signaling pathway (GO:0043122, GO:0007249, GO:0043123), the regulation of interleukin-12 production (GO:0032655, GO:0032615, GO:0032695, GO:0042090, GO:0045075, GO:0045084), the activation and positive regulation of the innate immune response (GO:0002757, GO:0002758, GO:0002253, GO:0050778, GO:0002218, GO:0002764, GO:0050776, GO:0045089, GO:0002684), and the MyD88-dependent toll-like receptor signaling pathway (GO:0002224, GO:0002755). In the other comparison group, the DElncRNA target genes were significantly enriched in 180 GO terms (*P* < 0.05) (Additional file 8: Table S8–2). Innate immunity-related GO terms were not found in this comparison group.

**Table 1:** Innate immunity-related GO terms which significantly regulated by the target genes of DElncRNAs

GO term ID	Term Descrption	Comparison group	*P* value
GO:0043122	regulation of I-kappaB kinase/NF-kappaB signaling	Mock-vs-PDCoV_12h	3.61E-03
GO:0002224	toll-like receptor signaling pathway	Mock-vs-PDCoV_12h	4.51E-03
GO:0007249	I-kappaB kinase/NF-kappaB signaling	Mock-vs-PDCoV_12h	4.95E-03
GO:0032655	regulation of interleukin-12 production	Mock-vs-PDCoV_12h	6.68E-03
GO:0032615	interleukin-12 production	Mock-vs-PDCoV_12h	9.44E-03
GO:0043123	positive regulation of I-kappaB kinase/NF-kappaB signaling	Mock-vs-PDCoV_12h	1.17E-02
GO:0002757	immune response-activating signal transduction	Mock-vs-PDCoV_12h	1.38E-02
GO:0032695	negative regulation of interleukin-12 production	Mock-vs-PDCoV_12h	1.61E-02
GO:0042090	interleukin-12 biosynthetic process	Mock-vs-PDCoV_12h	1.61E-02
GO:0045075	regulation of interleukin-12 biosynthetic process	Mock-vs-PDCoV_12h	1.61E-02
GO:0045084	positive regulation of interleukin-12 biosynthetic process	Mock-vs-PDCoV_12h	1.61E-02
GO:0002758	innate immune response-activating signal transduction	Mock-vs-PDCoV_12h	1.70E-02
GO:0002218	activation of innate immune response	Mock-vs-PDCoV_12h	2.75E-02
GO:0002755	MyD88-dependent toll-like receptor signaling pathway	Mock-vs-PDCoV_12h	3.07E-02
GO:0045089	positive regulation of innate immune response	Mock-vs-PDCoV_12h	4.11E-02

Moreover, the target genes were also annotated against the KEGG database (https://www.kegg.jp/kegg/kegg1.html) [35], and enrichment analysis was performed. Sixty-one and 28 KEGG pathways were significantly enriched in Mock-vs-PDCoV_12h and PDCoV_12h-vs-PDCoV_24h, respectively (*P* < 0.05) (Fig. 6B, Additional file 8: S8–3, S8–4). Notably, the target genes in Mock-vs-PDCoV_12h were significantly enriched in multiple innate immunity-related pathways, including the TNF signaling pathway (ko04668), Toll-like receptor signaling pathway (ko04620), NOD-like receptor signaling pathway (ko04621), RIG-I-like receptor signaling pathway (ko04622), mTOR signaling pathway (ko04150), and NF-kappa B signaling pathway (ko04064) (Table 2). The innate immunity-related pathway TNF signaling pathway (ko04668) was significantly enriched in another comparison group (Table 2).

**Table 2:** Innate immunity-related KEGG pathways which significantly regulated by the target genes of DElncRNAs

Pathway ID	Pathway	Comparison group	*P* value
ko04668	TNF signaling pathway	Mock-vs-PDCoV_12h	6.24E-06
ko04620	Toll-like receptor signaling pathway	Mock-vs-PDCoV_12h	2.06E-04
ko04621	NOD-like receptor signaling pathway	Mock-vs-PDCoV_12h	1.29E-03
ko04622	RIG-I-like receptor signaling pathway	Mock-vs-PDCoV_12h	6.91E-03
ko04150	mTOR signaling pathway	Mock-vs-PDCoV_12h	1.98E-02
ko04064	NF-kappa B signaling pathway	Mock-vs-PDCoV_12h	2.36E-02
ko04668	TNF signaling pathway	PDCoV_12h-vs-PDCoV_24h	6.35E-03

### Function and pathway enrichment analysis of targets of DEmiRNAs

**Fig. 7:**
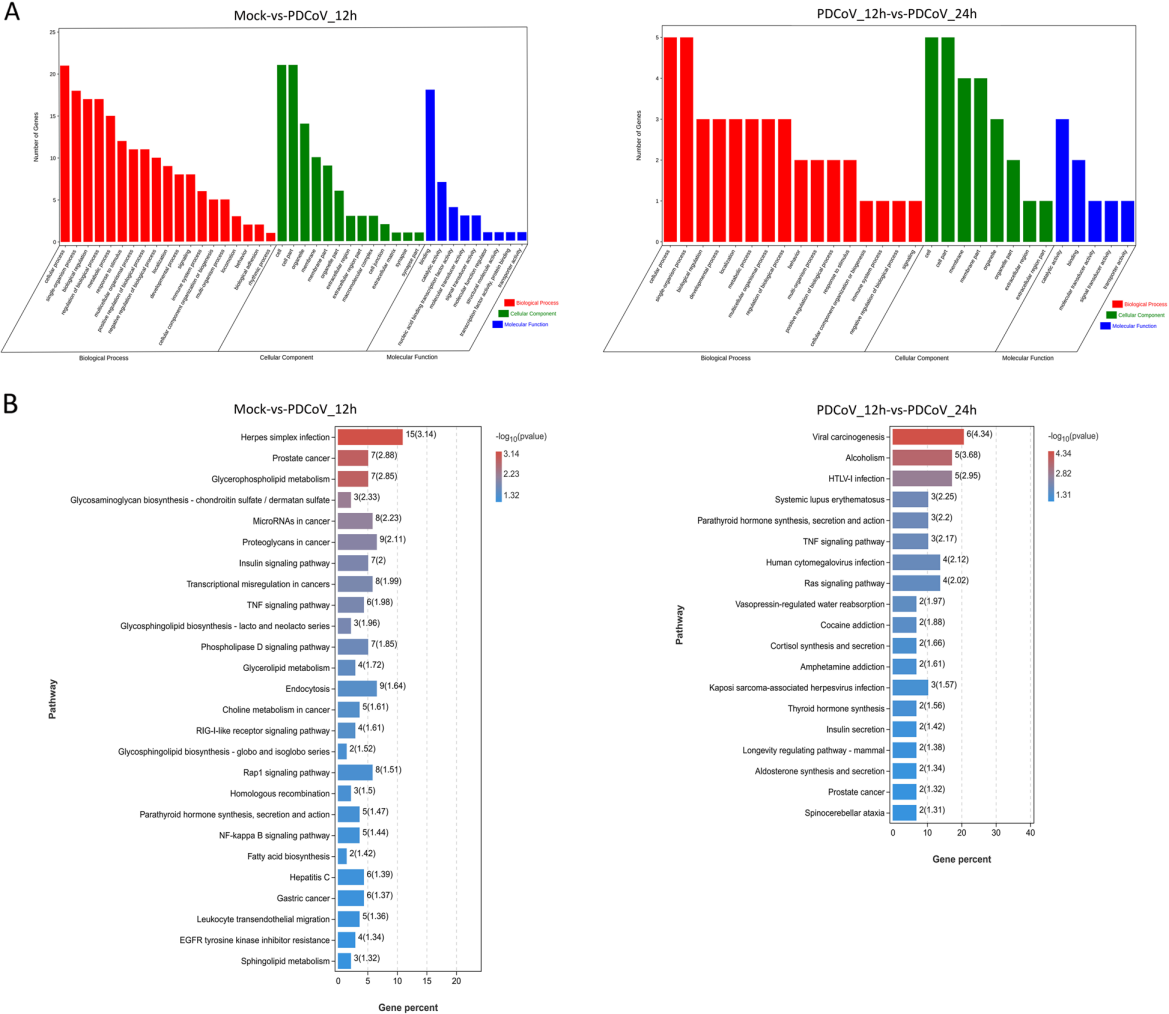
GO function classification and KEGG pathway enrichment results of the target genes of DEmiRNAs in Mock-vs-PDCoV_12h and PDCoV_12h-vs-PDCoV_24h. **A** GO function classification of the target genes of DEmiRNAs in the two comparison groups. **B** The top 30 KEGG pathways which enriched in the two comparison groups

Target genes of DEmiRNAs which were annotated against the GO database (http://geneontology.org/) (Fig. 7A). Most of the annotated functions belonged to *Biological Process.* Among them, the functions affected by PDCoV in the two comparison groups were also almost the same, which included the regulation of biological processes of cellular immunity in response to external stimulation (immune system process and response to stimulus). The target genes of DEmiRNAs in Mock-vs-PDCoV_12h were significantly enriched in 279 GO terms (*P* < 0.05) (Additional file 9: Table S9–1). Among these, GO terms relevant to the innate immune response were enriched, such as regulation of the toll-like receptor 7 signaling pathway (GO:0034154, GO:0034155, GO:0034157), regulation of the production of interleukin-12 (GO:0032655 and GO:0032615), response to interferon-alpha (GO:0035455) and regulation of I-kappaB kinase/NF-kappaB signaling (GO:0043122, GO:0007249, GO:0007250, GO:0032088) (Table 3). In another comparison group, the DEmiRNA target genes were significantly enriched in 80 GO terms (*P* < 0.05) (Additional file 9: Table S9–2). Innate immunity-related pathways were not found in this comparison group.

**Table 3:** Innate immunity-related GO terms which significantly regulated by the target genes of DEmiRNAs

GO term ID	Term Descrption	Comparison group	*P*-value
GO:0032655	regulation of interleukin-12 production	Mock-vs-PDCoV_12h	1.84E-02
GO:0034154	toll-like receptor 7 signaling pathway	Mock-vs-PDCoV_12h	1.98E-02
GO:0034155	regulation of toll-like receptor 7 signaling pathway	Mock-vs-PDCoV_12h	1.98E-02
GO:0034157	positive regulation of toll-like receptor 7 signaling pathway	Mock-vs-PDCoV_12h	1.98E-02
GO:0032615	interleukin-12 production	Mock-vs-PDCoV_12h	2.19E-02
GO:0043122	regulation of I-kappaB kinase/NF-kappaB signaling	Mock-vs-PDCoV_12h	3.36E-02
GO:0007249	I-kappaB kinase/NF-kappaB signaling	Mock-vs-PDCoV_12h	3.79E-02
GO:0007250	activation of NF-kappaB-inducing kinase activity	Mock-vs-PDCoV_12h	3.91E-02
GO:0032088	negative regulation of NF-kappaB transcription factor activity	Mock-vs-PDCoV_12h	3.91E-02
GO:0035455	response to interferon-alpha	Mock-vs-PDCoV_12h	3.91E-02

In addition, 26 and 19 KEGG pathways were significantly enriched in Mock-vs-PDCoV_12h and PDCoV_12h-vs-PDCoV_24h, respectively (*P* < 0.05) (Fig. 7B, Additional file 9: Table S9–3 and S9–4). Among them, three innate immunity-related pathways were enriched in Mock-vs-PDCoV_12h, including the TNF signaling pathway (ko04668), RIG-I-like receptor signaling pathway (ko04622), and NF-kappa B signaling pathway (ko04064) (Table 4). The innate immunity-related pathway TNF signaling pathway (ko04668) was enriched in PDCoV_12h-vs-PDCoV_24h (Table 4).

**Table 4:** Innate immunity-related KEGG pathways which significantly regulated by the target genes of DEmiRNAs

Pathway ID	Pathway	Comparison group	*P* value
ko04668	TNF signaling pathway	Mock-vs-PDCoV_12h	1.05E-02
ko04622	RIG-I-like receptor signaling pathway	Mock-vs-PDCoV_12h	2.46E-02
ko04064	NF-kappa B signaling pathway	Mock-vs-PDCoV_12h	3.66E-02
ko04668	TNF signaling pathway	PDCoV_12h-vs-PDCoV_24h	6.82E-03

### Construction of competing endogenous RNA networks centred on innate immunity-related miRNAs

**Fig. 8:**
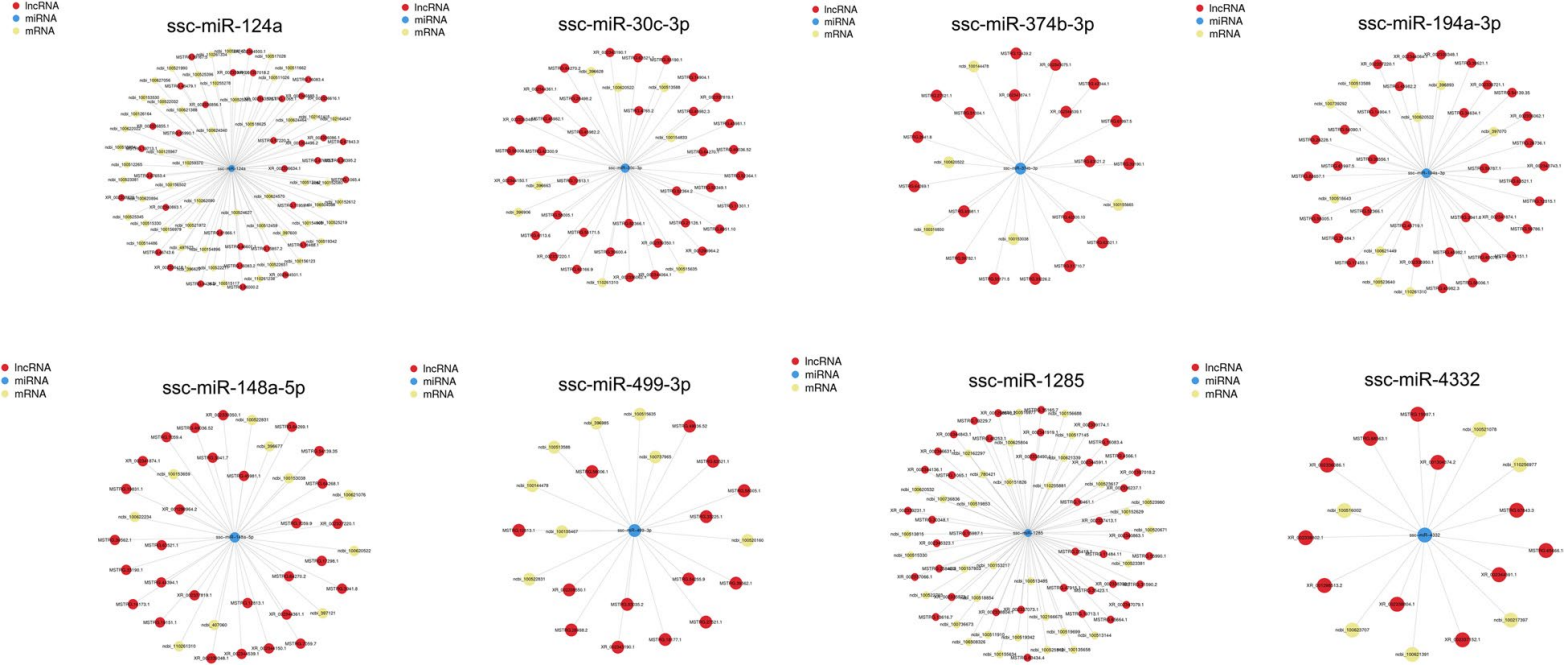
CeRNA regulatory network centred on innate immunity-related miRNAs in Mock-vs-PDCoV_12h. The top 8 miRNAs that had the highest connectivity of innate immunity-related target genes were selected for representation

In addition to lncRNAs and miRNAs regulating their target mRNAs, lncRNAs can also act as endogenous miRNA sponges, resulting in a reduction in the negative regulatory effect of miRNAs on mRNAs [36]. Thereby, competing endogenous RNA (ceRNA) networks among innate immunity-related lncRNAs, miRNAs and mRNAs centred on miRNAs were established using Cytoscape (v3.7.1) [37]. The top 8 miRNAs that had the highest connectivity of innate immunity-related target genes were selected for representation (Fig. 8).

### Functional validation of miRNA for the regulation of PDCoV replication

**Fig. 9:**
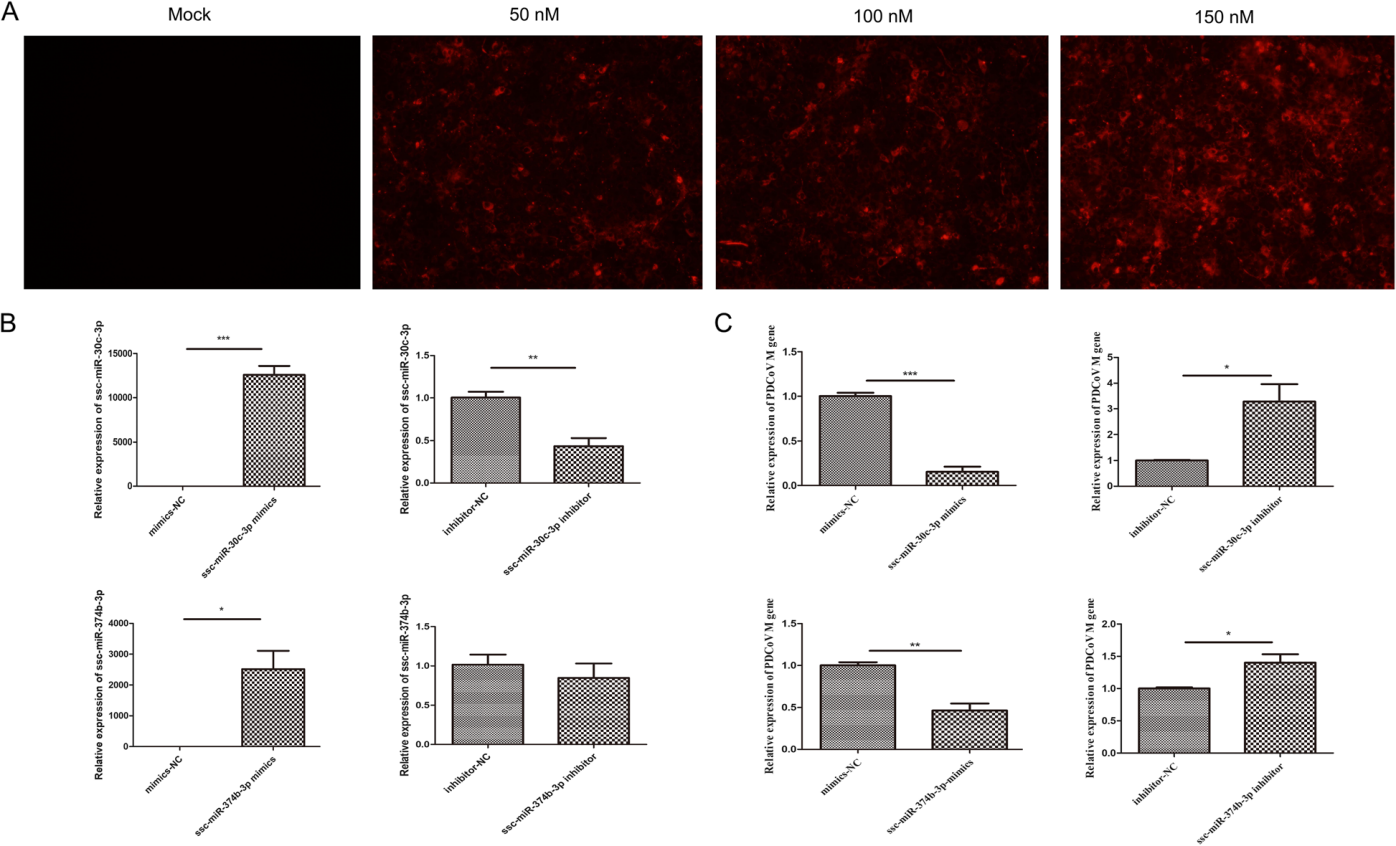
Ssc-miR-30c-3p and ssc-miR-374b-3p inhibited the replication of PDCoV. **A** The transfection of miRNA mimics fluorescence control at concentrations of 50 nM, 100 nM and 150 nM. After 24 h, the cells transfected with miRNA mimics fluorescence control were presented in red fluorescence. **B** The relative expression of ssc-miR-30c-3p and ssc-miR-374b-3p in the cells transfected with mimics or inhibitor as well as the controls of ssc-miR-30c-3p and ssc-miR-374b-3p. **C** Overexpression of ssc-miR-30c-3p or ssc-miR-374b-3p inhibited the replication of PDCoV. Inhibition of the expression of ssc-miR-30c-3p or ssc-miR-374b-3p increased the replication of PDCoV. Data are shown as mean ± SEM, n = 3. *P < 0.05, **P < 0.01, ***P < 0.001

We observed that in the Mock-vs-PDCoV_12h comparison group, more innate immunity-related functions and pathways were regulated by both DElncRNAs and DEmiRNAs than in the other comparison group. The results indicated that more ncRNAs might have participated in the innate immune evasion of PDCoV in the cells at 12 hpi than in the cells at 24 hpi. We selected two miRNAs (ssc-miR-30c-3p and ssc-miR-374b-3p) from the miRNAs that had high connectivity with innate immunity-related target genes in Mock-vs-PDCoV_12h and had higher alterations in expression in both Mock and PDCoV_12h to explore their regulatory role in PDCoV replication. IPEC-J2 cells were first transfected with the miRNA mimic fluorescence control at concentrations of 50 nM, 100 nM and 150 nM, and 150 nM, which had the highest fluorescence in the microscopic observation results, was selected for subsequent experiments (Fig. 9A). The results showed that overexpression of ssc-miR-30c-3p or ssc-miR-374b-3p significantly inhibited the replication of PDCoV (Fig. 9B and C). In contrast, inhibiting the expression of ssc-miR-30c-3p or ssc-miR-374b-3p significantly enhanced PDCoV replication (Fig. 9B and C).

## Discussion

High-throughput sequencing can help us obtain the gene expression profile of virus-infected host cells. Based on the analysis of the sequencing data, we can further identify the host cell response induced by virus infection to further understand the pathogenic mechanism of the virus. In this study, the replication of PDCoV was enhanced in IPEC-J2 cells with increasing infection time. Thus, we selected the cells infected with PDCoV at three infection time points during the proliferation of PDCoV as sequencing samples to explore the pathogenic mechanism of PDCoV from a new perspective by integrated analysis of RNA and small RNA sequencing data. All of the expressed lncRNAs, miRNAs and mRNAs in PDCoV-infected cells at three different postinfection times were identified. Subsequently, DElncRNAs, DEmiRNAs and DEmRNAs that were strongly affected by PDCoV during infection were screened from Mock-vs-PDCoV_12h and PDCoV_12h-vs-PDCoV_24h. The targets of the DElncRNAs and DEmiRNAs were then predicted and further used to perform functional and pathway enrichment analyses. These results represent the functions and pathways that were primarily regulated by key lncRNAs and miRNAs in IPEC-J2 cells during PDCoV infection.

Moreover, we found a large number of innate immunity-related DEncRNA target genes, and multiple innate immunity-related functions and pathways were enriched in the two comparison groups, which indicated that ncRNAs might play an important role in regulating the innate immune response during PDCoV infection. In Mock-vs-PDCoV_12h, the number of innate immunity-related target genes of ncRNAs and the enriched innate immunity-related functions and pathways were higher than those in the other comparison group, which indicated that the regulation of ncRNAs in the innate immune response in IPEC-J2 cells occurs mainly at 12 hpi compared to 24 hpi. From the GO functions and KEGG pathways commonly shown in the enrichment results of the DElncRNA and DEmiRNA target genes in Mock-vs-PDCoV_12h, we found that innate immunity-related functions that were related to the I-kappa B kinase/NF-kappaB signaling pathway, interleukin-12 production, and the toll-like receptor signaling pathway, and innate immunity-related pathways, including the RIG-I-like receptor signaling pathway, NF-kappa B signaling pathway and TNF signaling pathway, were regulated by both DElncRNAs and DEmiRNAs. PDCoV has evolved several strategies to escape the host cell innate immune response to complete replication. PDCoV infection disrupts the activation of the transcription factors IRF3 and NF-κB as well as the association of IKKε with both TRAF3 and IRF3 to inhibit IFN-β production [11, 16]. Additionally, the cleavage of NF-κB essential modulator (NEMO), a critical constituent of the IKK complex (IKK-α, IKK-β, and IKK-γ), caused by PDCoV NSP5 impedes the synthesis of type I IFN [13]. Among the genes involved in the process by which PDCoV escapes the innate immune response, IRF3 is involved in the RIG-I-like signaling pathway, and NF-κB, TRAF3, IKKε and NEMO are all involved in the RIG-I-like signaling pathway and NF-kappa B signaling pathway (map04622 and map04064 in KEGG database: https://www.kegg.jp/kegg/kegg1.html). These pathways are consistent with the pathways commonly regulated by both DElncRNAs and DEmiRNAs in our current study.

lncRNA expression profiles in intestinal tissues of piglets at 2, 4 and 11 days postinfection (dpi) by PDCoV were investigated using RNA sequencing analysis by Tang et al. [20]. Due to the differences in sequencing samples and postinfection times, the number of lncRNAs identified in the present study was much higher than that reported in the study by Tang et al. Additionally, the study did not identify significantly enriched innate immunity-related functions and pathways, which might be because the functions and pathways involved in tissue samples are more complex than those in cell samples, and the postinfection time points were different from those in the current study. In another study, Liu et al. investigated the expression profile of lncRNAs in ST cells, which are porcine testicular cells and are susceptible to PDCoV infection [21]. The postinfection time of the study (11 hpi) was similar to the postinfection time in the current study (12 hpi), but the reported viral infection dose was different from that of the current study. Even so, the number of identified lncRNAs in the study was similar to that in the current study. In Liu’s study, the target genes of transcriptome-wide lncRNAs were used to perform GO function and KEGG pathway enrichment analyses. This may be why the innate immunity-related pathways enriched in the study were not exactly the same as those in the present study. Even so, the innate immunity-related NF-kappaB signaling pathway and TNF signaling pathway were enriched in Liu’s study and were included in the enriched pathways regulated by both DElncRNAs and DEmiRNAs in our current study.

In this study, we also constructed competing endogenous RNA (ceRNA) networks centred on innate immunity-related miRNAs. Among the 8 vital miRNAs represented in this study, some have been identified and are known as crucial regulators in the viral pathogenic process. For example, miR-124a is able to markedly inhibit porcine reproductive and respiratory syndrome virus (PRRSV) replication in PAM cells by directly targeting CD163, a potential receptor or factor for PRRSV infection [38]. miR-148a-5p regulates duck Tembusu virus replication by targeting SOCS1, a negative regulator of the cytokine signaling cascade that plays an important role in the immune response [39]. The functional roles of other miRNAs, including miR-30c-3p, miR-374b-3p, miR-194a-3p, miR-499–3p, miR-4332 and miR-1285, in regulating the viral pathogenic innate immune response are still unclear. Additionally, we found that ssc-miR-30c-3p or ssc-miR-374b-3p inhibited the replication of PDCoV in IPEC-J2 cells. The results indicated that these two miRNAs may participate in PDCoV infection by regulating the innate immune response. However, the detailed regulatory mechanism needs to be further explored. It is worth noting that miR-30c-3p and miR-30a-5p are both members of the miR-30 family. It has been found that ssc-miR-30a-5p is involved in the innate immune evasion of TGEV [40]. SOCS1 and SOCS3, which are negative regulators of the innate immunity-related Jak-STAT signaling pathway, are target genes of ssc-miR-30a-5p. Ssc-miR-30a-5p enhances the cellular innate immune response by directly targeting SOCS1 and SOCS3. TGEV inhibits the expression of endogenous ssc-miR-30a-5p in host cells by regulating IRE1 α, resulting in an increase in the expression of SOCS1 and SOCS3, thereby inhibiting the antiviral activity of type I IFN and promoting TGEV replication. However, whether ssc-miR-30c-3p affects the replication of TGEV or PDCoV through regulatory mechanisms similar to those of ssc-miR-30a-5p remains to be determined. This hypothesis needs to be further studied.

## Conclusions

In this study, we determined the miRNA, lncRNA, and mRNA expression profiles of PDCoV-infected IPEC-J2 cells at three postinfection times by small RNA and RNA sequencing. The ncRNAs, including lncRNAs and mRNAs, and their potential target mRNAs as well as the functions and pathways that are primarily regulated during PDCoV infection in IPEC-J2 cells were screened from two different comparison groups. A series of innate immunity-related findings were obtained, and the regulatory role of two miRNAs in PDCoV replication was investigated. Our data provide valuable information for further exploring the specific role of ncRNAs in the pathogenic mechanism of PDCoV.

## Materials and methods

### Cell culture

IPEC-J2 (Jennio Biotech, China), which was used for PDCoV infection and sequencing after infection, was maintained in Dulbecco’s modified Eagle’s medium (DMEM, Gibco, USA) supplemented with 1% penicillin–streptomycin (100 units/ml of penicillin and 100 μg/ml streptomycin) and 10% foetal bovine serum (FBS) (Gibco, USA). Cells were incubated at 37 °C with 5% CO_2_.

### PDCoV infection

PDCoV strain TJ_1_ was previously isolated from small intestine specimens of piglets showing diarrheal syndromes on a farm of Tianjin and stored at Tianjin Institute of Animal Husbandry and Veterinary Medicine, Tianjin Academy of Agricultural Sciences, Tianjin, China [41]. PDCoV at a final MOI of 1.0 was used for adsorption in monolayer IPEC-J2 cells in an incubator at 37 °C in 5% CO_2_ for 2 h, followed by replacement with new cell culture medium consisting of DMEM supplemented with 1% pancreatin (Sigma-Aldrich, USA). At 0, 12, and 24 hpi, the CPE of the PDCoV-infected IPEC-J2 cells was observed, and the cells were harvested for RNA isolation. For each condition, three individual replicates were set up. IPEC-J2 cells at 0, 12 and 24 hpi were harvested in triplicate for further miRNA sequencing analysis.

### Total RNA isolation and quantification of the PDCoV M gene

Total RNA of PDCoV-infected IPEC-J2 cells at 0, 12, and 24 hpi was extracted using a TRIzol reagent kit (Invitrogen, USA) according to the manufacturer’s instructions. RNA integrity (RIN) was measured using an Agilent 2100 Bioanalyzer (Agilent Technologies, Germany), while the purity and concentration of total RNA were measured using a NanoDrop ND-2000 spectrophotometer (Thermo Fisher Scientific, USA). PDCoV cDNA was obtained using the PrimeScript™ RT reagent Kit (Perfect Real Time) (Takara Biomedical Technology, China) on an Applied Biosystems™ Veriti™ Dx 96-well Fast Thermal Cycler (Thermo Fisher Scientific, USA). Determination of the copy number of the PDCoV M gene was performed using the GoTaq® Probe qPCR Master Mix reagent (Promega, USA) according to the detection method established in our previous study [42]. The reactions were performed on an ABI7500 StepOnePlus Real-Time PCR System (Thermo Fisher Scientific, USA). Sequences of forward primer, reverse primer and probe are listed in Additional file 10: Table S10.

### Library construction and sequencing

For RNA sequencing, coding RNA and ncRNA were first screened by removing rRNA from total RNA. The obtained RNAs were were randomly disrupted to obtain short fragments with a Magnesium RNA Fragmentation Module kit (NEB) following the manufacturer’s instructions. Single-strand cDNAs were generated based on these fragment templates and random hexamers, and double-strand cDNAs were generated based on single-strand cDNA templates purified by a QIAquick PCR Purification Kit (Thermo Fisher Scientific, USA). The termini of the cDNA sequences were repaired, and poly (A) and sequencing adapters were added. Then, the second strands were enzymatically degraded by UNG uracil-N-glycosylase. Subsequently, these cDNAs were screened using AMPure XP beads (Beckman, USA) according to sizes and amplified by PCR to obtain the libraries. RNA sequencing was performed using an Illumina Novaseq6000 instrument (Gene Denovo Biotechnology Co., China).

For small RNA sequencing, RNA samples were purified by polyacrylamide gel electrophoresis (PAGE), and the RNA molecules with a length range of 18–30 nt were enriched. Subsequently, sequencing adapters were ligated to the 5′ and 3′ termini of the RNA. The ligation products were reverse transcribed and further amplified by PCR. The PCR products with a size range of 140–160 bp were enriched to generate small RNA sequencing libraries and eventually deep sequenced on an Illumina Novaseq6000 platform (Gene Denovo Biotechnology Co., China).

### Analysis of RNA-Seq data

High-quality clean reads were first acquired from raw reads, and then mapped to the porcine reference genome *Sus scrofa* 11.1 by HISAT2. Based on the results of genome mapping, the transcripts were assembled by StringTie according to a previous study [43], and then further mapped to *Sus scrofa* 11.1. The known lncRNAs and mRNAs were obtained by comparing the assembled transcripts with annotated lncRNAs and mRNAs in the reference genome. Then, novel transcripts with length ≥ 200 bp and number of exons ≥2 were further retained from the transcripts that mapped to the reference genome and were located in intergenic regions. Then CNCI and CPC2 software were used to predict the protein-coding potential of novel transcripts. Novel lncRNAs were predicted by removing the novel transcripts that did not pass the protein-coding score test (CPC score < 0, CNCI score < 0), and the remaining transcripts were novel mRNAs. The expression of lncRNAs and mRNAs was calculated by fragments per kilobase of transcript per million mapped reads (FPKM) using RSEM software .

### Analysis of small RNA-Seq data

High-quality clean reads were first screened from raw reads by removing the low-quality reads and adapter sequences. Then, the clean reads in a size range from 18 to 30 nt were further screened against the GenBank database (Release 209.0) [44] and Rfam (11.0) database [45] to remove small nucleolar RNA, small nuclear RNA, rRNA, tRNA, repeat sequences, exon and intron sequences. Eventually, the existed miRNAs were identified by searching all of the clean reads in the miRbase database [46] (Release 22). The known miRNAs that were still not included in the miRBase database were identified by aligning clean reads to those existed miRNAs of other species which included in miRBase. To distinguish with the existed miRNAs, x (−x) and y (−y) were used to represent the known miRNAs from the 5′ (−5p) and 3′ (−3p) arms of the miRNA precursor in this study. To identify the novel miRNAs, all of the unannotated clean reads were aligned with the swine reference genome (NCBI genome database: *Sus scrofa* 11.1) and the novel miRNAs were screened according to the results of hairpin structure prediction which performed by miReap software [47]. For gene identification, clean reads were screened from raw reads obtained from RNA sequencing by removing low-quality reads and reads with adapters or unknown nucleobases. Subsequently, the expressed genes and novel genes were identified by genome mapping. Briefly, clean reads obtained from RNA sequencing were mapped to a swine reference genome (NCBI genome database: *Sus scrofa* 11.1), which was performed by HISAT software following the instructions of the software website (http://www.ccb.jhu.edu/software/hisat/index.shtml). The miRNA expression level was computed and normalized to transcripts per million (TPM) [48].

### Screening of differentially expressed lncRNAs, mRNAs and miRNAs

The expression analysis of lncRNA, mRNA and miRNA in the two comparison groups were performed using DEGseq2 (http://www.bioconductor.org/packages/release/bioc/html/DESeq2.html) and edgeR (http://www.bioconductor.org/packages/release/bioc/html /edgeR.html). lncRNAs and mRNAs with a false discovery rate (FDR) value < 0.05 and a │log2 (fold change) │ > 1 and miRNAs with a *P* < 0.05 and a │log2 (fold change) │ > 1 were identified as differentially expressed ncRNAs or mRNAs.

### Prediction of lncRNA and miRNA target genes

The potential target genes of DElncRNAs involved in *cis*- and *trans*-regulatory effects were predicted. We screened the mRNAs located 10 kb upstream and downstream of lncRNAs as *cis* target genes. The *trans* target genes were predicted by correlation analysis between lncRNAs and mRNAs. According to the FPKM values, the Pearson correlation coefficient between the lncRNAs and the mRNAs was calculated, and the threshold for positive correlation was set to Pearson correlation > 0.999.

Three software programs, TargetScan (Version 7.0), RNAhybrid (Version 2.1.2) and miRanda (Version 3.3a), were used to predict the targets of miRNAs. The genes that had reliable binding sites (minimum free energy (MFE) levels < − 10 kcal/mol, *P* values < 0.05) to miRNAs and commonly existed in 3 prediction results were considered to be credible targets of miRNAs. Targets involved in the innate immune response were annotated to InnateDB (https://www.innatedb.ca/).

### RT-qRCR validation

Six miRNAs were randomly selected for RT-qPCR analysis using Bulge-loop™ miRNA RT-qPCR Primer Sets (RiboBio Inc., China) according to the manufacturer’s instructions. The primers for miRNAs and internal standard U6 were designed by RiboBio Inc. (China), and the sequences are covered by a patent. The RT-qPCR examination of lncRNAs and mRNAs was carried out using TB Green® *Premix Ex Taq*™ II (Tli RNaseH Plus) (Takara Biomedical Technology, China) following the manufacturer’s instructions. Primers for lncRNAs and mRNAs were designed and are listed in Additional file 10: Table S10. The RT-qPCRs were performed on an ABI7500 StepOnePlus Real-Time PCR System (Thermo Fisher Scientific, USA) and run in duplicate. U6 was used as the internal reference gene for miRNA relative expression validation, and GAPDH was used as the internal reference gene for lncRNA and mRNA relative expression validation. The relative expression level of each miRNA, lncRNA and mRNA was calculated using the 2^−ΔΔCt^ method [49, 50].

### Function and pathway enrichment analysis

To investigate the function of lncRNAs and miRNAs in IPEC-J2 cells, which were primarily influenced by PDCoV infection, GO functional enrichment analysis and KEGG pathway enrichment analysis of the predicted targets of DElncRNAs and DEmiRNAs were further performed by using of the GOseq R package and KOBAS software (3.0) [51]. The significantly enriched functional GO terms and pathways are represented in this study (*P* < 0.05).

### Construction of competing endogenous RNA networks

Innate immunity-related lncRNAs, miRNAs and mRNAs were chosen for analysis. lncRNA-miRNA interactions were predicted by miRanda. Based on the results of the innate immunity-related target genes of DElncRNAs and DEmiRNAs as well as the lncRNA-miRNA interactions, visualization of the lncRNA-miRNA-mRNA interaction network was constructed using Cytoscape software (http://www.cytoscape.org/download.php).

### Functional validation of miRNA for the regulation of PDCoV replication

Monolayer IPEC-J2 cells were transfected with miRNA mimic fluorescence controls under different concentrations using Lipofectamine 3000 (Thermo Fisher Scientific, USA). After 24 h, the fluorescence of the cells was observed by fluorescence microscopy (Olympus Corporation, Japan). Subsequently, monolayer IPEC-J2 cells were transfected with mimics and inhibitor as well as the controls of ssc-miR-30c-3p and ssc-miR-374b-3p, respectively, at a concentration of 150 nM. After 24 h, PDCoV at a final MOI of 1.0 was then used for adsorption in the transfected IPEC-J2 cells in an incubator at 37 °C in 5% CO_2_. The cells were respectively harvested at 24 h after transfection and at 12 hpi, and then the total RNA of each sample was extracted by TRIzol. miRNA RT product and cDNA was respectively obtained using the riboSCRIPT Reverse Transcription Kit (RiboBio Inc., China) and the PrimeScript™ RT reagent Kit (Perfect Real Time) (Takara Biomedical Technology, China) in an Applied Biosystems™ Veriti™ Dx 96-well Fast Thermal Cycler (Thermo Fisher Scientific, USA). The relative expression of ssc-miR-30c-3p and ssc-miR-374b-3p in the cells transfected with mimics or inhibitor as well as the controls of ssc-miR-30c-3p and ssc-miR-374b-3p were detected by RT-qPCR using Bulge-loop™ miRNA RT-qPCR Primer Sets (RiboBio Inc., China) according to the manufacturer’s instructions. The primers for miRNAs and internal standard U6 were designed by RiboBio Inc. (China), and the sequences are covered by a patent. The relative expression of the PDCoV M gene in different transfection groups was detected by RT-qPCR using TB Green® *Premix Ex Taq*™ II (Tli RNaseH Plus) (Takara Biomedical Technology, China). The RT-qPCRs were performed on an ABI7500 StepOnePlus Real-Time PCR System (Thermo Fisher Scientific, USA). Sequences of forward primer and reverse primer for RT-qPCR of PDCoV M gene are listed in Additional file 10: Table S10.

## Supplementary Information

**Additional file 1.** Overview of the RNA sequencing data.

**Additional file 2.** Overview of the small RNA sequencing data.

**Additional file 3.** Differentially expressed lncRNAs, miRNAs and mRNAs.

**Additional file 4.** Prediction results of the target genes of DElncRNAs.

**Additional file 5.** Prediction results of the target genes of DEmiRNAs.

**Additional file 6.** Innate immunity-related target genes of DElncRNAs.

**Additional file 7.** Innate immunity-related target genes of DEmiRNAs.

**Additional file 8.** Enrichment analysis result of DElncRNAs' target genes.

**Additional file 9.** Enrichment analysis result of DEmiRNAs' target genes.

**Additional file 10.** Primers used for qRT-PCR examination in this study.

## Data Availability

The data of identification and integrated analysis of circRNAs and miRNAs has not been published, so the sequencing raw data cannot be disclosed temporarily. The raw data of sequencing can be obtained by contacting the corresponding author (E-mail: yanmh81971@126.com). The datasets generated during this study are included in the article and its additional files.
